# How can technology enhance cognitive behavioral therapy: the case of pediatric obsessive compulsive disorder

**DOI:** 10.1186/s12888-017-1377-0

**Published:** 2017-06-23

**Authors:** Lidewij H. Wolters, Vivian op de Beek, Bernhard Weidle, Norbert Skokauskas

**Affiliations:** Norwegian University of Science and Technology (NTNU), Faculty of Medicine, Regional Centre for Child and Youth Mental Health and Child Welfare (RKBU Central Norway), Klostergata 46, 7030 Trondheim, Norway

**Keywords:** Obsessive compulsive disorder, OCD, Children, Adolescents, Cognitive behavioral therapy, tCBT, Technology, Internet, Smartphone application, Videoconferencing

## Abstract

Many children with mental health disorders do not receive adequate treatment due to the uneven dissemination of resources, and other barriers to treatment. In the case of pediatric obsessive compulsive disorder treatment progress is also hindered by partial or non-response to treatment in addition to poor compliance. This debate paper focuses on new technologies as a potential vehicle to address the challenges faced by traditional treatment, with special reference to cognitive behavioral therapy for pediatric obsessive compulsive disorder. We discuss the achievements and challenges that previous studies have faced, debate ways to overcome them, and we offer specific suggestions for further research in the area.

## Background

Mental health disorders are highly prevalent, already among children and adolescents.^1^ Up to one third of children suffer from a mental health disorder at some point during their lifetime [1, 2]. These disorders are associated with reduced quality of life, and with educational and work problems, and they involve high societal and personal costs [3]. Despite the increase in evidence-based treatments, only 10–30% of children with mental health disorders seeking help receive adequate treatment [4–6]. This can be largely attributed to several barriers that limit the availability, accessibility, and acceptability of evidence-based treatments, such as a shortage of qualified therapists, and an insufficient dissemination of evidence-based care [7, 8]. Barriers to care may be further increased by logistic and financial obstacles, job- or school-related restrictions, and shame and stigma [8, 9].

Pediatric obsessive compulsive disorder (OCD) is a relatively common [4, 10–12], severe, and debilitating condition, characterized by obsessions and compulsions, and associated with high rates of comorbidity [13]. If untreated, OCD symptoms often persist into adulthood [14], and lead to substantial impairments in family, academic and social functioning, and to a reduced quality of life [15–17]. Cognitive behavioral therapy (CBT) is the first-line treatment for pediatric OCD [18], and its effectiveness has been extensively demonstrated [19–21]. However, treatment for OCD is hampered by several problems [9, 22].

First of all, average improvement rates are limited and there are large individual differences in treatment effect [23, 24]. For partial and non-responders to CBT a combination of CBT and selective serotonin reuptake inhibitors (SSRIs) is recommended [25]. However, some recent studies cast doubt on the additional value of medication. Although the POTS trial showed that, on average, combined treatment (CBT plus SSRI) was superior to CBT monotherapy for children with OCD, this effect could be attributed to the results of only one site. No superior effect of the combined treatment over CBT monotherapy was found for the other main site [23]. Subsequently, Storch et al. compared the effectiveness of CBT plus pill placebo with CBT plus an SSRI, in which CBT was delivered by experienced therapists. In this study, no evidence was found for a superior effect of the combined treatment over CBT monotherapy [26]. In line with these findings, Skarphedinsson et al. found that continuing CBT monotherapy was just as effective as adding an SSRI to continued CBT for initial non-responders to CBT monotherapy [27]. Furthermore, the use of medication entails several disadvantages, such as possible adverse effects, a heightened chance of relapse by discontinuation, and unknown effects in the long term [25, 28]. As a result, many parents have reservations about the safety and long-term benefit of medication use [6]. Taken together, this highlights the need for alternative options to the addition of medication to improve treatment for pediactric OCD.

Second, there are organizational and practical barriers to treatment for OCD. Although CBT is the treatment of choice, the availability of this treatment is limited, particularly in remote areas [4, 29]. There is a shortage of experienced therapists [29–31], and there are often long waiting lists for treatment [32]. Furthermore, studies in adult OCD patients revealed that CBT was often poorly implemented [9, 33]. A substantial proportion of participants reported having received pharmacological treatments, or various psychotherapeutic treatments other than CBT, such as ‘talk-therapy’, supportive therapy, psychodynamic therapy and EMDR [9, 33]. If the use of CBT was reported this treatment often did not meet criteria for state-of-the-art CBT [33]. In the case of pediatric OCD, the picture seems no better. Even in relatively prosperous countries like the United Kingdom and Norway, a lack of the availability of adequate treatment has been reported [4, 29]. An epidemiological study in the United Kingdom showed that only 12% of children with OCD identified in the survey had contacted child specialist services [4]. In addition, a nationwide survey among clinicians in Norway revealed that only 62% of the respondents applied CBT for pediatric OCD, with CBT being mainly cognitive oriented and E/RP often missing. Furthermore, most clinicians were inexperienced in treating OCD, and expressed a need for training opportunities [29].

Further hampering insufficient dissemination of adequate treatment, distances from qualified therapists may limit the accessibility of CBT [9, 34]. In addition, practical problems with scheduling, treatment associated costs, and disorder-specific symptoms that restrict mobility, can further limit accessibility to treatment [9, 31, 35].

Shame and stigma, patients’ beliefs about treatment, such as reluctance to engage in exposure exercises, and low confidence because of prior treatment failures, are other factors that restrain patients from seeking treatment [9, 30, 35, 36]. Consequently, too few patients get adequate help despite the strong evidence in favor of an effective treatment [4, 29].

### Technology empowered CBT

Modern technologies provide an opportunity to overcome at least some of these challenges. In this paper we use the term technology empowered CBT (tCBT) to refer to CBT based interventions integrating technology varying from basic online bibliotherapy to online self-help therapy, therapist-supported computerized CBT, smartphone applications (apps), traditional CBT delivered via telephone or videoconferencing, and combinations of these forms.

Recently, several initiatives have been taken to develop tCBT programs for OCD, and preliminary evidence shows that overall these programs yield positive effects [31, 37, 38]. Results from a meta-analysis of randomized controlled trials of tCBT programs for mainly adult OCD patients showed large effect sizes, and a stable effect over at least one month post-treatment. tCBT was found to be superior to control conditions (waitlist and relaxation), without a significant difference in efficacy between tCBT and traditional therapist-delivered CBT [37]. tCBT programs however vary significantly in format, duration, intensity, length, and effect size, and evidence for their effectiveness is limited by the small number of trials, small sample sizes, and an emphasis on the adult population. This stresses the need for further research, especially in children.

The encouraging, albeit preliminary, results for tCBT programs for OCD, and the challenges faced by current treatment, pose several questions: how can new technologies help to make treatment more accessible, more user-friendly, and more cost-effective?; can tCBT be a vehicle for a more intensive and focused application of CBT principles?; how can we take advantage of the attractiveness of mobile technologies to children and offer an attractive treatment form and space positively affecting treatment motivation, adherence, and effect? The main aim of this paper is to discuss if and how technology can enhance CBT for pediatric OCD, guided by previous relevant studies and making suggestions for future interventions.

## Discussion

### How can technology be used to improve the current situation of CBT for OCD?

In this section, we will discuss how tCBT can address several challenges currently faced by CBT, i.e., limited efficacy, insufficient availability and accessibility to adequate treatment, logistic barriers to treatment, and stigma.

tCBT can be delivered in a variety of formats, serving different goals. There are stand-alone programs, without any therapist contact, that can serve as the first step in a stepped care approach for patients with mild complaints, or provide an acceptable treatment option for patients who are reluctant to engage in face-to-face treatment [39, 40]. These programs may make treatment more cost-effective, improve the availability and accessibility of CBT, and meet issues of shame, stigma and convenience.

Other tCBT programs use technologies as the main intervention form supplemented with support of a therapist [37, 38]. Comparable to stand-alone programs, these programs offer the opportunity to improve the availability, accessibility, and cost-effectiveness of CBT, while offering more convenience for patients, as treatment can be (mainly) completed at home and therapist resources are used sparingly.

Technologies can also be used as an augmentation strategy to traditional CBT. In these formats CBT is delivered by a therapist, and supported or enriched by the use of technologies [32, 41]. This could address issues of accessibility and effectiveness of CBT by increasing treatment adherence and motivation.

Finally, technologies can serve as a medium to deliver therapist-administered CBT to patients at a distance, using tele- or videoconferencing [38, 42]. These formats improve the accessibility of treatment for patients who would otherwise not have access to treatment due to various geographical or transportation factors.

### Concerns about tCBT

Despite the advantages that tCBT programs may entail, the development of these programs has raised concerns. A few studies have reported on clinicians’, children’s, and parents’ attitudes towards tCBT [43–46]. The most frequently reported concerns by clinicians were about drop-out rates, the suitability of tCBT for severe complaints, the therapeutic alliance, the availability of professional support, and standardized programs that are not tailored to individual needs [43, 45]. In a survey among a small sample of children and parents using child and adolescent mental health services, children reported both an interest in, and a reluctance for computerized therapy [44]. Almost half of the children preferred to talk with a therapist rather than using a self-help computer program which was preferred by 9% of the sample. Concerns of parents included the lack of face-to-face contact with a therapist, safety issues related to internet usage, the negative effects of computer games (such as the addiction to games and social isolation), and children getting access to poor quality or harmful information when using the internet. Benefits of computerized (self-help) treatments identified by parents were related to reducing shame and stigma, opportunities for independent help seeking, access to good quality information, attractiveness of and familiarity with computer usage, and opportunities for peer support [44]. In general, clinicians’, children’s, and parents’ attitudes were generally positive and encouraging for the use of tCBT [43–45]. In line with this, an online survey among 129 adults with OCD showed that overall tCBT was rated as an acceptable potential form of treatment. A minority of the respondents feared potential disadvantages, including preference for face-to-face therapist contact, concerns that one’s problems would be too severe for tCBT, and concerns about the lack of non-verbal communication [46]. For the interpretation of the above results it is important to note that the majority of respondents in these studies had little knowledge about and no experience with tCBT [44, 45], which implies that reports mainly express expectations and are not based on experiences with tCBT. In addition, the studies reported on attitudes toward tCBT interventions in general, and did not distinguish between different formats. Nevertheless, clinicians’ as well as patients’ attitudes and believes about tCBT may affect the implementation of tCBT programs, and deserve attention.

Another type of concern is that the effectiveness of tCBT programs is not yet well established. Earlier studies provided that all of the earlier discussed tCBT formats can be effective in decreasing OCD symptoms, and that the overall effectiveness of tCBT might be comparable to therapist-administered CBT for OCD [31, 37, 38]. However, the reported effect sizes of tCBT programs for OCD are mainly based on adult samples, have varied substantially [31, 37], and results from a meta-analysis showed a non-significant trend suggesting that on average therapist-administered CBT was slightly more effective than tCBT [37].

Unfortunately, so far the literature does not provide specific information about which formats might be more effective than others. Nevertheless, findings from recent systematic reviews [31, 38], and published reports on tCBT for pediatric OCD, may provide clues to improve tCBT programs for OCD and to address the challenges of current treatment, taking concerns into account. A computerized search of Pubmed and PsycInfo databases, and a manual search within the reference lists of relevant papers identified nine studies reporting on tCBT interventions for pediatric OCD (see Table 1 for an overview of these studies). Most of these studies were recently published and therefore not included in the available meta-analysis and reviews. Below we discuss suggestions for future tCBT programs based on present findings, starting with a look at the recent systematic reviews on tCBT for mainly adult OCD, followed by studies on pediatric OCD.

**Table 1:** tCBT programs for pediatric OCD

Authors	Type of intervention	Age group	Content intervention	Duration of intervention	Study design	Study outcome	Aimed contribution to current treatment
Rees et al. (2015) [59]	CBT self-help program no therapist contact	Adolescents 12–18 years	A website offered self-guided treatment including interactive elements, personalized feedback, and a reminder system. The treatment contains E/RP (main component), cognitive restructuring, coping with stress, and family accommodation. The program consists of eight modules with separate content for adolescents and for parents.	8 weeks	Open trial	Under study	Increasing treatment availability and accessibility, and improving cost-effectivity (stepped care model)
Lenhard et al. (2014; 2017) [60, 61]	Web-based CBT reduced therapist contact	Adolescents 12–17 years	The program (12 chapters for adolescents and 5 for parents) contains educative texts, interactive elements, animations, films and exercises, addressing psychoeducation, E/RP, cognitive elements, relapse prevention, family accommodation and parental coping strategies. Participants can have regular contact with a therapist through e-mails, phone calls and standardized forms. The treatment is supported by a smartphone app offering the possibility to add and edit exposure tasks and set reminders for ERP.	12 weeks	RCT *N* = 67	Results of the RCT showed a moderate effect size (CYBOCS effect size *d* = 0.69) for the web-based CBT compared to a waiting list. Average therapist time was 17.5 min per week per participant. Almost half of the adolescents reported that they were satisfied with the treatment, 50% were satisfied most of the time but would have liked face-to-face contact with a therapist occasionally, and 4% would have preferred face-to-face treatment.	Increasing treatment availability, accessibility, and improving cost-effectivity
Whiteside et al. (2014) [34]	CBT smartphone application reduced therapist contact	Children and adolescents (not further specified)	App that can be used both as stand-alone CBT intervention with minimal therapist contact in cases of milder symptoms, and as adjunct to face-to-face CBT in cases of more severe OCD combined with geographical barriers. The app contains 3 modules: assessment, psychoeducation, and treatment. The treatment module guides patients through the E/RP. Patients can track their progress over time.	Not reported	Case examples *N* = 2	Results indicate that both applications of the app can be effective. The app appeared to encourage treatment adherence and to facilitate exposure exercises between sessions. Detailed information via the app about exposure exercises at home was helpful for treatment management.	Increasing treatment availability and accessibility, and improving cost-effectivity
Farrell et al. (2016) [36]	Video conferencing sessions after brief, intensive CBT full therapist contact	Adolescents 11–16 years	The treatment package consisted of a face-to-face psychoeducation session and two intensive CBT sessions (three hours per session) with exposure as the main component, followed by three therapist-guided video conferencing sessions aimed at continuation of the exposure exercises and relapse prevention.	6 weeks	A multiple baseline controlled study *N* = 10	Results showed an overall reduction in OCD severity after treatment, and gains were maintained during a six months follow-up period. Eight of ten children were considered reliable improved	Increasing treatment accessibility, efficiency, and improving cost-effectivity
Storch et al. (2011) [50]	CBT delivered via video conferencing (w-CBT) full therapist contact	Children and adolescents 7–16 years	Therapist-guided, web-camera delivered CBT (14 sessions) based on the protocol used in POTS (2004), including psychoeducation, cognitive therapy, E/RP, and relapse prevention. Parents were instructed to coach E/RP exercises out of the therapist’s view. At least one parent attended the sessions with the child.	12 weeks	Preliminary RCT, w-CBT versus 4 weeks waitlist *N* = 31	w-CBT was found to be effective, and superior to the waiting list control. Average reduction in OCD severity (CYBOCS) was 56% for the w-CBT arm (pre- to post-treatment *d* = 1.09). Despite a slight significant increase of OC symptom severity, gains were generally maintained in a naturalistic 3-month follow-up for w-CBT.	Increasing treatment accessibility
Comer et al. (2014) [49]	CBT delivered via video conferencing full therapist contact	Young children (4–8 years)	Internet-delivered family-based CBT based on the protocol of Freeman & Garcia (2009), including externalizing OCD, E/RP, contingency management, parental accommodation, and relapse prevention. Parents are trained as coaches for their children and play a key role throughout the treatment. The program consists of 12 therapist-guided sessions with child and parents, and contains interactive elements and computer games.	14 weeks	Case series *N* = 5	All children completed the full treatment course. Effect size for within-subjects CY-BOCS changes was large (*d* = 2.54), although results showed individual differences in treatment effect; 60% no longer met diagnostic criteria for OCD at post-treatment. All mothers characterized the quality of services as excellent.	Increasing treatment accessibility
Turner et al. (2009; 2014) [53, 54]	Telephone delivered CBT (t-CBT) full therapist contact	Adolescents 11–18 years	Up to 14 weekly telephone CBT sessions. t-CBT consisting of psychoeducation, E/RP, cognitive interventions, and relapse prevention. Parents were involved (10 min parental discussion at the end of each treatment session).	Within 17 weeks	RCT, t-CBT compared to face-to-face CBT. *N* = 72	Results indicated that telephone delivered CBT was equally effective as face-to-face CBT until 6-month follow-up. Non-inferiority could not be established at 12-month follow-up. After t-CBT, 88% of the participants fulfilled the criterion for responder (≥ 35% CYBOCS reduction), and 59% for remission (CYBOCS ≤12). Participants reported to be satisfied with both interventions.	Increasing treatment accessibility

Note. *E/RP* Exposure with response prevention, *RCT* Randomized controlled trial

### What can we learn from recent systematic reviews?

First, more effective treatment programs are characterized by a better implementation of the exposure element of CBT [31, 38]. Although (almost) all programs include exposure, the degree and way in which exposure is incorporated varied, ranging from vicarious exposure on the screen to active exposure in real-life situations, and from following a self-help program to therapist-guided exposure using a web-camera [31]. Results from a study of Greist et al. showed that the number of completed real-life exposure exercises correlated positively with OCD symptom reduction [47]. These findings suggest that an intensive and focused application of exposure may be a core element of effective tCBT programs.

Second, incorporating therapist contact may lead to better treatment effects [31, 38]. In a study of Kenwright et al. evaluating computer-aided self-help for OCD, scheduled therapist contact contributed to reduced drop-out rates and to enhanced compliance compared with therapist contact on demand only [48].

A point of concern is the high drop-out rate in some studies, specifically for stand-alone programs [31, 38]. Possible explanations are the lack of therapist contact [48], and the inflexibility of fully-automated programs. Therapists can support and motivate patients leading to more engagement and treatment adherence, and can assist in solving upcoming problems, while stand-alone programs do not have the possibility to flexibly respond to personal situations, characteristics of patients, and unexpected problems [31, 38]. These results point to the additional value of therapist contact, and also to treatment programs that can, at least to some degree, address unique needs of individual patients.

### What can we learn from studies in tCBT for pediatric OCD?

As shown in Table 1, a variety of tCBT programs for pediatric OCD have been developed. Although preliminary, the present studies provide encouraging results in improving the availability and accessibility of tCBT and reducing treatment related costs and burden by offering programs that can be completed (partly) at home. Furthermore, to address the issue of limited availability of CBT in remote areas, programs have been developed based on teleconferencing and videoconferencing instead of in-office meetings with a therapist.

In line with the findings above, two case examples in the study of Whiteside et al. point to the importance of therapist support to help families to maintain focus on treatment, and to be able to provide assistance by exposure exercises when needed. Another suggestion following from this study, is that tools offering space for personalized items may be preferred over pre-made items, as pre-made items may not always fit [34].

The study of Whiteside et al. provides preliminary evidence that technologies can augment therapist-delivered CBT. In a case example of a boy with severe OCD who lived too far away to frequently visit a therapist, an app was successfully used to facilitate exposure exercises at home between face-to-face treatment sessions, allowing for fewer frequent visits to the therapist. The app also generated data about the conductance of exposure exercises at home. These data provide therapists with information that can be used to solve possible problems in an early stage, and can be used by researchers to increase knowledge about effective ingredients of treatment [34]. In addition, findings show that technology can be used to extend exposure from the therapist’s office to the patient’s daily life where the symptoms naturally occur [34, 49].

A study of Farrell et al. suggests that technology based interventions can fulfill a role in the maintenance and continuation of treatment effects after therapist-delivered CBT [36].

Some points of concern regarding technology-based approaches are also reported. Storch et al. pointed out that although overall experiences with their web-camera based approach were positive, therapists reported some difficulty establishing a therapeutic relationship, particularly with more oppositional children [50]. Challenges in handling disruptive behavior during web-camera sessions were also reported by Comer et al. In addition, therapists experienced more difficulties with reading body language during web-camera sessions compared to face-to-face sessions [49]. On the other hand, there is some evidence for CBT via videoconferencing in adults with OCD suggesting that a therapeutic relationship can be successfully established [51, 52].

In conclusion, tCBT programs can address at least some treatment barriers for pediatric OCD, although it must be borne in mind that most findings are preliminary, and that more robust study designs and larger samples are needed to extend these findings. Future research will have to address several issues. First of all, more information is needed about which factors can make tCBT treatments more effective and which treatment forms are most effective and for whom. We have discussed the available studies in an attempt to find some clues to answer this question. Second, recently developed tCBT programs for pediatric OCD primarily aim to improve the availability, accessibility, and cost-effectiveness of treatment, and do not address the problem that CBT is not sufficiently effective for all patients. A reasonable next step would be to use technologies to develop an *enhanced* treatment for pediatric OCD, addressing the issues of partial- and non-response, noncompliance, and drop-out from traditional CBT, alongside improving access and availability and making treatment more cost-effective. To address the limitations of previous studies and the challenges of the current situation, we discuss below an enhanced CBT (eCBT) concept for pediatric OCD.

### How can technology be used to enhance treatment for pediatric OCD?

To address the issues of limited effectiveness and treatment drop-out from (t)CBT, the eCBT program needs a focused and intensive application of exposure [31, 38, 47], and should be therapist-guided to help families setting up adequate exposure exercises, to enhance treatment compliance, to motivate patients to adhere to the exposure exercises, to solve possible problems, and to address individual needs of patients [31, 34, 38, 48]. Therefore, therapist-administered CBT could be augmented with an app which has a monitoring, scaffolding and motivating function to support the exposure exercises between treatment sessions and encourage treatment compliance [34, 41]. The app might prompt patients to engage in exposure exercises, to support recording of homework and assessments, to encourage positive behavior, and to personalize treatment by allowing for individualized content and settings. Data on these activities gathered by the app provides the therapist with actual information that can be used to solve problems directly, set up new exposure exercises, and optimize the therapeutic process. Signs of noncompliance can be monitored regularly and early steps can be taken immediately to address problems. The app might allow for contact with the therapist between treatment sessions in order to prevent unnecessary stagnation in progress.

Furthermore, a partly home-based approach that could be accomplished by delivering CBT with exposure as the core element, through videoconferencing based sessions at home combined with in-office face-to-face therapist contact, might overcome barriers to care, and may offer more convenience for the children and their families through reducing travelling time, costs, and stigma [36, 49, 50, 53, 54]. Videoconferencing sessions may further add to in-office CBT as delivering interventions in settings in which the problems occur may increase ecologically validity and encourage the generalization of principles from therapy-related to natural settings [34]. It also addresses the concerns and preferences of children and parents by incorporating visual contact with the therapist [43, 46]. Finally, videoconferencing sessions offer the opportunity to easily intensify treatment, if required, by extra web-camera guided exposure exercises between sessions, which may further reduce non-response. To address concerns regarding therapeutic alliance, videoconferencing sessions can be combined with face-to-face sessions [34].

Modern technologies, such as apps, can also contribute to the attractiveness of treatment for children, which may further encourage treatment compliance and prevent treatment drop-out [31, 38, 55]. The use of technological applications fits easily into the lives of today’s children, and may offer a user-friendly tool [41, 44]. For example, video clips of children with OCD can be used for psychoeducation, which may reduce shame, and applications can incorporate interactive elements for administrating exposure exercises, tracking treatment progress, rewarding positive behavior, and building a relapse prevention plan which may have a motivational effect alongside personalizing treatment. An app can also be used for generating psychological data (for example in vivo assessments of mood and symptoms), as well as physiological data (for example by connecting the app to a wristband measuring physiological indicators for stress during exposure) in a user-friendly way, which can serve clinical as well as research purposes, both of which may contribute to the improvement of treatment [32, 41].

Finally, for a successful implementation of such a program, training for users can be offered as many therapists may not have experience with tCBT programs, and concerns of clinicians towards this form of treatment [43, 45], and how tCBT can address these concerns will have to be discussed with them. For example, when therapists are concerned about the suitability of tCBT for severe or difficult-to-treat symptoms, treatment can be intensified by extra videoconferencing sessions between the regular sessions to guide exposure sessions at home. Regarding concerns about drop-out rates, a motivating app supporting homework exercises for clients and providing feedback about treatment progress to therapists may improve treatment compliance and prevent pre-term treatment drop out.

In addition, where applicable, the concerns of children and parents related to tCBT will have to be addressed by the therapist before the start of treatment [44]. Safety and privacy related to the usage of technological applications need to be ensured. Finally, acceptability and feasibility studies of tCBT treatments are needed to further improve tCBT treatment packages.

If such an enhanced CBT program has been proven to be effective in pediatric OCD, the model could be modified and applied to other mental health disorders, as many of the treatment barriers and limitations discussed in this paper are not restricted to OCD [8]. An app which supports exercises at home and thereby encourages treatment compliance could also be used as an augmentation to CBT for other disorders, for example supporting exposure exercises for anxiety disorders, behavioral activation for depression, E/RP or habit reversal for tic disorders, and healthy eating patterns in eating disorders. In addition, videoconferencing sessions to guide exposure sessions at home could be incorporated into exposure based treatments for a variety of disorders. Overall, the use of modern technologies, such as apps, may be attractive for all young people and could therefore offer a useful medium for the delivery of CBT to a range of disorders [41, 55–58].

## Conclusion

Technological innovations offer a unique opportunity to address limitations associated with traditional treatment such as access, suitability, expense, and stigma. Furthermore, the ever-increasing integration of sophisticated electronic media into young people’s lives seems to offer a good opportunity to use it also for therapeutic purposes. In the case of pediatric OCD, preliminary results for CBT programs integrating technology are encouraging, but several challenges need to be addressed by enhancing tCBT programs with the focus on non-response, non-compliance, and preterm treatment drop-outs. This may contribute to a more effective treatment for pediatric OCD, and could offer a framework for other disorders too.

## Abbreviations

CBT: Cognitive behavioral therapy
eCBT: Enhanced cognitive behavioral therapy
E/RP: Exposure with response prevention
tCBT: Technology empowered cognitive behavioral therapy
OCD: Obsessive compulsive disorder
RCT: Randomized controlled trial
SSRI: Selective serotonin reuptake inhibitor

## Footnotes

1 On behalf of the readability of this paper we use the term ‘children’ to refer to both children and adolescents in the subsequent text. When children and adolescents are distinguished, this is indicated in the text.

## References

[1] Merikangas KR, Nakamura EF, Kessler RC (2009). Epidemiology of mental disorders in children and adolescents. Dialogues Clin Neurosci.

[2] Polanczyk GV, Salum GA, Sugaya LS, Caye A, Rohde LA (2015). Annual Research Review: A meta-analysis of the worldwide prevalence of mental disorders in children and adolescents. J Child Psychol Psychiatry.

[3] Olesen J, Gustavsson A, Svensson M, Wittchen HU, Jönsson B (2012). on behalf of the CDBE2010 study group and the European Brain Council. The economic cost of brain disorders in Europe. Eur J Neurol.

[4] Heyman I, Fombonne E, Simmons H, Ford T, Meltzer H, Goodman R (2001). Prevalence of obsessive—compulsive disorder in the British nationwide survey of child mental health. Br J Psychiatry.

[5] Schwartz C, Schlegl S, Kuelz AK, Voderholzer U (2013). Treatment-seeking in OCD community cases and psychological treatment actually provided to treatment-seeking patients: A systematic review. Journal of Obsessive-Compulsive and Related Disorders.

[6] Rutter M, Bishop D, Pine D, Scott S, Stevenson J, Taylor E (2008). Rutter's child and adolescent psychiatry.

[7] Comer JS (2015). Introduction to the special series: Applying new technologies to extend the scope and accessibility of mental health care. Cogn Behav Pract.

[8] Wang J (2006). Perceived barriers to mental health service use among individuals with mental disorders in the Canadian general population. Med Care.

[9] Marques L, LeBlanc NJ, Weingarden HM, Timpano KR, Jenike M, Wilhelm S (2010). Barriers to treatment and service utilization in an internet sample of individuals with obsessive–compulsive symptoms. Depress Anxiety.

[10] Douglass HM, Moffitt TE, Dar R, McGee R, Silva P (1995). Obsessive-compulsive disorder in a birth cohort of 18-year-olds: Prevalence and predictors. Journal of the American Academy of Child & Adolescent Psychiatry.

[11] Flament MF, Whitaker A, Rapoport JL, Davies M, Berg CZ, Kalikow K (1988). Obsessive compulsive disorder in adolescence: An epidemiological study. J Am Acad Child Adolesc Psychiatry.

[12] Rapoport JL, Inoff-Germain G, Weissman MM, Greenwald S, Narrow WE, Jensen PS (2000). Childhood obsessive-compulsive disorder in the NIMH MECA study: Parent versus child identification of cases. J Anxiety Disord.

[13] Geller DA, Biederman J, Faraone S, Agranat A, Cradock K, Hagermoser L (2001). Developmental aspects of obsessive compulsive disorder: findings in children, adolescents, and adults. J Nerv Ment Dis.

[14] Stewart SE, Geller DA, Jenike M, Pauls D, Shaw D, Mullin B (2004). Long-term outcome of pediatric obsessive–compulsive disorder: A meta-analysis and qualitative review of the literature. Acta Psychiatr Scand.

[15] Piacentini J, Bergman RL, Keller M, McCracken J (2003). Functional impairment in children and adolescents with obsessive-compulsive disorder. J Child Adolesc Psychopharmacol.

[16] Valderhaug R, Ivarsson T (2005). Functional impairment in clinical samples of Norwegian and Swedish children and adolescents with obsessive-compulsive disorder. Eur Child Adolesc Psychiatry.

[17] Weidle B, Jozefiak T, Ivarsson T, Thomsen PH (2014). Quality of life in children with OCD with and without comorbidity. Health Qual Life Outcomes.

[18] National Institute for Health and Care Excellence (NICE). Obsessive–compulsive disorder and body dysmorphic disorder: treatment. Clinical Guideline [CG31]. London: NICE; 2005. 39480980

[19] O'Kearney RT, Anstey K, Von Sanden C, Hunt A (2010). Behavioural and cognitive behavioural therapy for obsessive compulsive disorder in children and adolescents (Review). Cochrane Database Syst Rev.

[20] Sanchez-Meca J, Rosa-Alcazar AI, Iniesta-Sepulveda M, Rosa-Alcazar A (2014). Differential efficacy of cognitive-behavioral therapy and pharmacological treatments for pediatric obsessive-compulsive disorder: A meta-analysis. J Anxiety Disord.

[21] Skarphedinsson G, Hanssen-Bauer K, Kornør H, Heiervang ER, Landrø NI, Axelsdottir B (2014). Standard individual cognitive behaviour therapy for paediatric obsessive–compulsive disorder: A systematic review of effect estimates across comparisons. Nord J Psychiatry.

[22] Torres AR, Prince MJ, Bebbington PE, Bhugra DK, Brugha TS, Farrell M (2007). Treatment seeking by individuals with obsessive-compulsive disorder from the British psychiatric morbidity survey of 2000. Psychiatr Serv.

[23] The Pediatric OCD Treatment Study (POTS) Team (2004). Cognitive-behavior therapy, sertraline, and their combination for children and adolescents with obsessive-compulsive disorder: The pediatric OCD treatment study (POTS) randomized controlled trial. JAMA.

[24] Torp NC, Dahl K, Skarphedinsson G, Thomsen PH, Valderhaug R, Weidle B (2015). Effectiveness of cognitive behavior treatment for pediatric obsessive-compulsive disorder: Acute outcomes from the Nordic Long-term OCD Treatment Study (NordLOTS). Behav Res Ther.

[25] Geller DA, March J, Walter HJ, Bukstein OG, Benson RS, Chrisman A (2012). Practice parameter for the assessment and treatment of children and adolescents with obsessive-compulsive disorder. J Am Acad Child Adolesc Psychiatry.

[26] Storch EA, Bussing R, Small BJ, Geffken GR, McNamara JP, Rahman O (2013). Randomized, placebo-controlled trial of cognitive-behavioral therapy alone or combined with sertraline in the treatment of pediatric obsessive–compulsive disorder. Behav Res Ther.

[27] Skarphedinsson G, Weidle B, Thomsen P, Dahl K, Torp N, Nissen J (2015). Continued cognitive-behavior therapy versus sertraline for children and adolescents with obsessive–compulsive disorder that were non-responders to cognitive-behavior therapy: a randomized controlled trial. Eur Child Adolesc Psychiatry.

[28] Storch EA, Larson MJ, Muroff J, Caporino N, Geller D, Reid JM (2010). Predictors of functional impairment in pediatric obsessive-compulsive disorder. J Anxiety Disord.

[29] Valderhaug R, Gunnar Götestam K, Larsson B (2004). Clinicians’ views on management of obsessive–compulsive disorders in children and adolescents. Nord J Psychiatry.

[30] Braddick F, Carral V, Jenkins R, Jané-Llopis E (2009). Child and adolescent mental health in Europe: Infrastructures, policy and programmes.

[31] Herbst N, Voderholzer U, Stelzer N, Knaevelsrud C, Hertenstein E, Schlegl S (2012). The potential of telemental health applications for obsessive–compulsive disorder. Clin Psychol Rev.

[32] Lind C, Boschen MJ, Morrissey S (2013). Technological advances in psychotherapy: Implications for the assessment and treatment of obsessive compulsive disorder. J Anxiety Disord.

[33] Stobie B, Taylor T, Quigley A, Ewing S, Salkovskis PM (2007). “Contents may vary”: A pilot study of treatment histories of OCD patients. Behav Cogn Psychother.

[34] Whiteside SPH, Ale CM, Vickers Douglas K, Tiede MS, Dammann JE (2014). Case examples of enhancing pediatric OCD treatment with a smartphone application. Clinical Case Studies.

[35] Goodwin R, Koenen KC, Hellman F, Guardino M, Struening E (2002). Helpseeking and access to mental health treatment for obsessive-compulsive disorder. Acta Psychiatr Scand.

[36] Farrell LJ, Oar EL, Waters AM, McConnell H, Tiralongo E, Garbharran V (2016). Brief intensive CBT for pediatric OCD with E-therapy maintenance. J Anxiety Disord.

[37] Dèttore D, Pozza A, Andersson G (2015). Efficacy of technology-delivered cognitive behavioural therapy for OCD versus control conditions, and in comparison with therapist-administered CBT: meta-analysis of randomized controlled trials. Cogn Behav Ther.

[38] Lovell K, Bee P (2011). Optimising treatment resources for OCD: A review of the evidence base for technology-enhanced delivery. J Ment Health.

[39] Mataix-Cols D, Marks IM (2006). Self-help with minimal therapist contact for obsessive–compulsive disorder: a review. Eur Psychiatry.

[40] Moritz S, Wittekind CE, Hauschildt M, Timpano KR (2011). Do it yourself? Self-help and online therapy for people with obsessive-compulsive disorder. Curr Opin Psychiatry.

[41] Jones DJ, Anton M, Gonzalez M, Honeycutt A, Khavjou O, Forehand R (2015). Incorporating mobile phone technologies to expand evidence-based care. Cogn Behav Pract.

[42] Nelson E-L, Duncan AB (2015). Cognitive behavioral therapy using televideo. Cogn Behav Pract.

[43] Stallard P, Richardson T, Velleman S (2010). Clinicians' attitudes towards the use of computerized cognitive behaviour therapy (cCBT) with children and adolescents. Behav Cogn Psychother.

[44] Stallard P, Velleman S, Richardson T (2010). Computer use and attitudes towards computerised therapy amongst young people and parents attending child and adolescent mental health services. Child Adolesc Mental Health.

[45] Vigerland S, Ljótsson B, Bergdahl Gustafsson F, Hagert S, Thulin U, Andersson G (2014). Attitudes towards the use of computerized cognitive behavior therapy (cCBT) with children and adolescents: a survey among Swedish mental health professionals. Internet Interventions.

[46] Wootton BM, Titov N, Dear BF, Spence J, Kemp A (2011). The acceptability of internet-based treatment and characteristics of an adult sample with obsessive compulsive disorder: an internet survey. PLoS One.

[47] Greist JH, Marks IM, Baer L, Kobak KA, Wenzel KW, Hirsch MJ (2002). Behavior therapy for obsessive-compulsive disorder guided by a computer or by a clinician compared with relaxation as a control. J Clin Psychiatry.

[48] Kenwright M, Marks I, Graham C, Franses A, Mataix-Cols D (2005). Brief scheduled phone support from a clinician to enhance computer-aided self-help for obsessive-compulsive disorder: Randomized controlled trial. J Clin Psychol.

[49] Comer JS, Furr JM, Cooper-Vince CE, Kerns CE, Chan PT, Edson AL (2014). Internet-delivered, family-based treatment for early-onset OCD: A preliminary case series. Journal of clinical child and adolescent psychology: the official journal for the Society of Clinical Child and Adolescent Psychology, American Psychological Association, Division 53.

[50] Storch EA, Caporino NE, Morgan JR, Lewin AB, Rojas A, Brauer L (2011). Preliminary investigation of web-camera delivered cognitive-behavioral therapy for youth with obsessive-compulsive disorder. Psychiatry Res.

[51] Goetter EM, Herbert JD, Forman EM, Yuen EK, Thomas JG (2014). An open trial of videoconference-mediated exposure and ritual prevention for obsessive-compulsive disorder. J Anxiety Disord.

[52] Himle JA, Fischer DJ, Muroff JR, Van Etten ML, Lokers LM, Abelson JL (2006). Videoconferencing-based cognitive-behavioral therapy for obsessive-compulsive disorder. Behav Res Ther.

[53] Turner C, Heyman I, Futh A, Lovell K (2009). A pilot study of telephone cognitive-behavioural therapy for obsessive-compulsive disorder in young people. Behav Cogn Psychother.

[54] Turner CM, Mataix-Cols D, Lovell K, Krebs G, Lang K, Byford S (2014). Telephone cognitive-behavioral therapy for adolescents with obsessive-compulsive disorder: A randomized controlled non-inferiority trial. Journal of the American Academy of Child & Adolescent Psychiatry.

[55] Kenny R, Dooley B, Fitzgerald A (2016). Developing mental health mobile apps: Exploring adolescents' perspectives. Health Informatics J.

[56] Pennant ME, Loucas CE, Whittington C, Creswell C, Fonagy P, Fuggle P (2015). Computerised therapies for anxiety and depression in children and young people: a systematic review and meta-analysis. Behav Res Ther.

[57] Rooksby M, Elouafkaoui P, Humphris G, Clarkson J, Freeman R (2015). Internet-assisted delivery of cognitive behavioural therapy (CBT) for childhood anxiety: systematic review and meta-analysis. J Anxiety Disord.

[58] Vigerland S, Lenhard F, Bonnert M, Lalouni M, Hedman E, Ahlen J (2016). Internet-delivered cognitive behavior therapy for children and adolescents: A systematic review and meta-analysis. Clin Psychol Rev.

[59] Rees CS, Anderson RA, Finlay-Jones A (2015). OCD? Not Me! Protocol for the development and evaluation of a web-based self-guided treatment for youth with obsessive-compulsive disorder. BMJ Open.

[60] Lenhard F, Vigerland S, Andersson E, Rück C, Mataix-Cols D, Thulin U (2014). Internet-delivered cognitive behavior therapy for adolescents with obsessive-compulsive disorder: An open trial. PLoS One.

[61] Lenhard F, Andersson E, Mataix-Cols D, Rück C, Vigerland S, Högström J (2017). Therapist-guided, internet-delivered cognitive-behavioral therapy for adolescents with obsessive-compulsive disorder: a randomized controlled trial. Journal of the American Academy of Child & Adolescent Psychiatry.

